# The association between serum uric acid and the incidence of prediabetes and type 2 diabetes mellitus: The Rotterdam Study

**DOI:** 10.1371/journal.pone.0179482

**Published:** 2017-06-20

**Authors:** Niels van der Schaft, Adela Brahimaj, Ke-xin Wen, Oscar H. Franco, Abbas Dehghan

**Affiliations:** Department of Epidemiology, Erasmus University Medical Center, Rotterdam, the Netherlands; Universita degli Studi Magna Graecia di Catanzaro Scuola di Medicina e Chirurgia, ITALY

## Abstract

**Background:**

Limited evidence is available about the association between serum uric acid and sub-stages of the spectrum from normoglycaemia to type 2 diabetes mellitus. We aimed to investigate the association between serum uric acid and risk of prediabetes and type 2 diabetes mellitus.

**Methods:**

Eligible participants of the Rotterdam Study (n = 8,367) were classified into mutually exclusive subgroups of normoglycaemia (n = 7,030) and prediabetes (n = 1,337) at baseline. These subgroups were followed up for incident prediabetes (n = 1,071) and incident type 2 diabetes mellitus (n = 407), respectively. We used Cox proportional hazard models to determine hazard ratios (HRs) for incident prediabetes among individuals with normoglycaemia and incident type 2 diabetes mellitus among individuals with prediabetes.

**Results:**

The mean duration of follow-up was 7.5 years for incident prediabetes and 7.2 years for incident type 2 diabetes mellitus. A standard deviation increment in serum uric acid was significantly associated with incident prediabetes among individuals with normoglycaemia (HR 1.10, 95% confidence interval (CI) 1.01; 1.18), but not with incident type 2 diabetes mellitus among individuals with prediabetes (HR 1.07, 95% CI 0.94; 1.21). Exclusion of individuals who used diuretics or individuals with hypertension did not change our results. Serum uric acid was significantly associated with incident prediabetes among normoglycaemic women (HR 1.13, 95% CI 1.02; 1.25) but not among normoglycaemic men (HR 1.08, 95% CI 0.96; 1.21). In contrast, serum uric acid was significantly associated with incident type 2 diabetes mellitus among prediabetic men (HR 1.23, 95% CI 1.01; 1.48) but not among prediabetic women (HR 1.00, 95% CI 0.84; 1.19).

**Conclusions:**

Our findings agree with the notion that serum uric acid is more closely related to early-phase mechanisms in the development of type 2 diabetes mellitus than late-phase mechanisms.

## Introduction

Uric acid is generated during nucleotide and adenosine triphosphate (ATP) metabolism and comprises the end product of human purine metabolism.[1] We have previously demonstrated in a large population-based cohort study that elevated serum levels of uric acid are associated with increased risk of type 2 diabetes mellitus (DM) independently of other risk factors.[2] This association has since then been replicated in many other prospective studies and subsequent meta-analyses.[3–6] In addition, serum uric acid has been associated with various cardiovascular and metabolic conditions such as hypertension, obesity, heart failure and atrial fibrillation in large population-based studies.[7]

Prediabetes is a disorder of glucose homeostasis characterized by impaired glucose tolerance or impaired fasting glucose. These are both reversible stages of intermediate hyperglycaemia that provide an increased risk of type 2 DM.[8] Prediabetes can therefore be regarded as an important reversible stage that could lead to type 2 DM, and early identification of prediabetes might contribute to the prevention of type 2 DM. Despite its established association with incident type 2 DM, serum uric acid has not been studied extensively in relation to incident prediabetes in individuals with normoglycaemia or incident type 2 DM in individuals with established prediabetes.

Therefore, the objective of the present study is to determine whether serum uric acid is associated with incident prediabetes among normoglycaemic individuals and type 2 DM among prediabetic individuals. This study is performed within the framework of the Rotterdam Study, a large population-based prospective cohort study of participants aged 45 years and older.[9]

## Materials and methods

### The Rotterdam Study

The methodology of the Rotterdam Study has been outlined extensively elsewhere.[9] Briefly, the study initially consisted of 7,983 residents of the Ommoord district aged 55 years and over in the city of Rotterdam, the Netherlands (RS-I). Following extension of the cohort in 2000 (RS-II), when individuals who had become 55 years of age or moved into the district since the study start were added to the cohort, and 2006 (RS-III), when individuals aged 45–54 years also became eligible for participation, the total number of subjects was 14,926 by the end of 2008.[9] These participants undergo physical examinations at the Rotterdam Study research facility and home interviews every 3–4 years. Data is collected on health status, risk factors for various diseases common in the elderly, anthropometric characteristics, incident disease and cause-specific mortality.[9] The Medical Ethics Committee of the Erasmus Medical Centre Rotterdam and the review board of the Dutch Ministry of Health, Welfare and Sport have approved this population-based cohort study, and all participants have provided written informed consent. For the purposes of this analysis, we combined data from cohorts RS-I (using the third visit in 1997–1999 as baseline), RS-II (baseline visit 2000–2001) and RS-III (baseline visit 2006–2009) of the Rotterdam Study.

### Definition of type 2 diabetes mellitus, prediabetes and normoglycaemia

As per the Rotterdam Study protocol and WHO guidelines, type 2 DM was defined as having a fasting plasma glucose level ≥ 7.0 mmol/L, a non-fasting plasma glucose ≥ 11.1 mmol/L, the use of oral anti-diabetic medication or insulin, treatment by diet with type 2 DM as an indication and/or being registered by a general practitioner as having type 2 DM.[10, 11] Prediabetes was defined as a fasting plasma glucose level 6.0–6.9 mmol/L or a non-fasting plasma glucose level 7.7–11.1 mmol/L, in addition to absence of all type 2 DM criteria. Normoglycaemia was defined as a fasting plasma glucose level ≤ 6.0 mmol/L and absence of any of the above criteria for prediabetes and type 2 DM. Fasting blood samples were obtained by means of venipuncture at the Rotterdam Study research facility. The samples were stored at −80°C in 5 mL aliquots. Within one week of sampling, glucose levels were measured by means of the glucose hexokinase method.[12] All measurements were performed at the clinical chemistry laboratory of Erasmus Medical Center, Rotterdam.

### Measurement of serum uric acid

Serum uric acid was determined in non-fasting blood samples, centrifuged for 10 minutes at 3,000 RPM and then stored for one week at -20°C. Uric acid activity was determined using a Kone Diagnostica reagent kit and a Kone auto-analyser. After every 10 samples, 3 control samples were included to check calibration. If the average values of the control samples were not within 2.5% of the true value in each run of 100 samples, this run was repeated. Day-by-day variation had to be within 5% of this average value.

### Covariates

In our study, the following covariates are considered: age, sex, body mass index (BMI), smoking status, daily alcohol intake, total serum cholesterol, serum HDL cholesterol, systolic blood pressure, serum insulin, serum glucose, hypertension (defined as having a systolic blood pressure > 140 mmHg, a diastolic blood pressure > 100 mmHg or receiving blood-pressure lowering medication with “hypertension” as an indication), physical activity, use of diuretics and estimated glomerular filtration rate (eGFR). Data on serum glucose, total serum cholesterol, serum HDL-cholesterol, serum insulin, blood pressure and eGFR were obtained at baseline by means of venipuncture, performed during participants’ visits to the Rotterdam Study research facility. Anthropometric characteristics were also recorded at the Rotterdam Study research facility. eGFR was calculated using the Chronic Kidney Disease Epidemiology Collaboration (CKD-EPI) equation.[13] The disease status with respect to type 2 DM and prediabetes was ascertained through follow-up using general practitioners’ records and hospital discharge letters, collected as part of the Rotterdam Study. Physical activity was assessed at baseline by means of a modified version of the Zutphen Study Physical Activity Questionnaire and the LASA Physical Activity Questionnaire.[9] Metabolic equivalents of task (MET) hours per week were calculated based on time spent in light, moderate and vigorous activity. Data concerning the use of medication, alcohol consumption and smoking at baseline was obtained through Rotterdam Study home interviews and, for medication, consulting pharmacy dispensing records.

### Statistical analysis

To determine the association between serum uric acid and risk of incident prediabetes or incident type 2 DM, Cox proportional hazards regression was performed with serum uric acid as the primary dependent variable and either incident prediabetes or incident type 2 DM as the response variable. The timescale in these models is follow-up time in years from baseline to either of the clinical endpoints, death, loss-to-follow-up or January 1st, 2012. Models adjusted only for age, sex and cohort as well as multivariable-adjusted Cox models were designed. The confounders BMI, smoking status, daily alcohol intake, total serum cholesterol, serum HDL cholesterol, systolic blood pressure, serum insulin, serum glucose, hypertension status, physical activity, use of diuretics and eGFR, selected based on previous literature, were added to the models adjusted for age, sex and cohort incrementally. The covariates serum insulin level, serum glucose level, daily alcohol intake and physical activity were log-transformed in the analyses because they displayed non-normality. Non-linearity was accounted for by inclusion of polynomial terms in the regression models if necessary. Interaction of uric acid with age and sex was investigated by introducing the product of the variables age and sex with uric acid to the regression models. Five-fold multiple imputation was performed to reduce bias that could arise from missing values. The results of our analyses are presented as hazard ratios (HR) with corresponding 95% confidence intervals. A p-value < 0.05 was considered statistically significant. Analyses were performed using SPSS Statistics version 21 (IBM Corp., Armonk, New York, USA) and R version 3.2.4 (The R foundation for Statistical Computing, Vienna, Austria).

## Results

**Fig 1:**
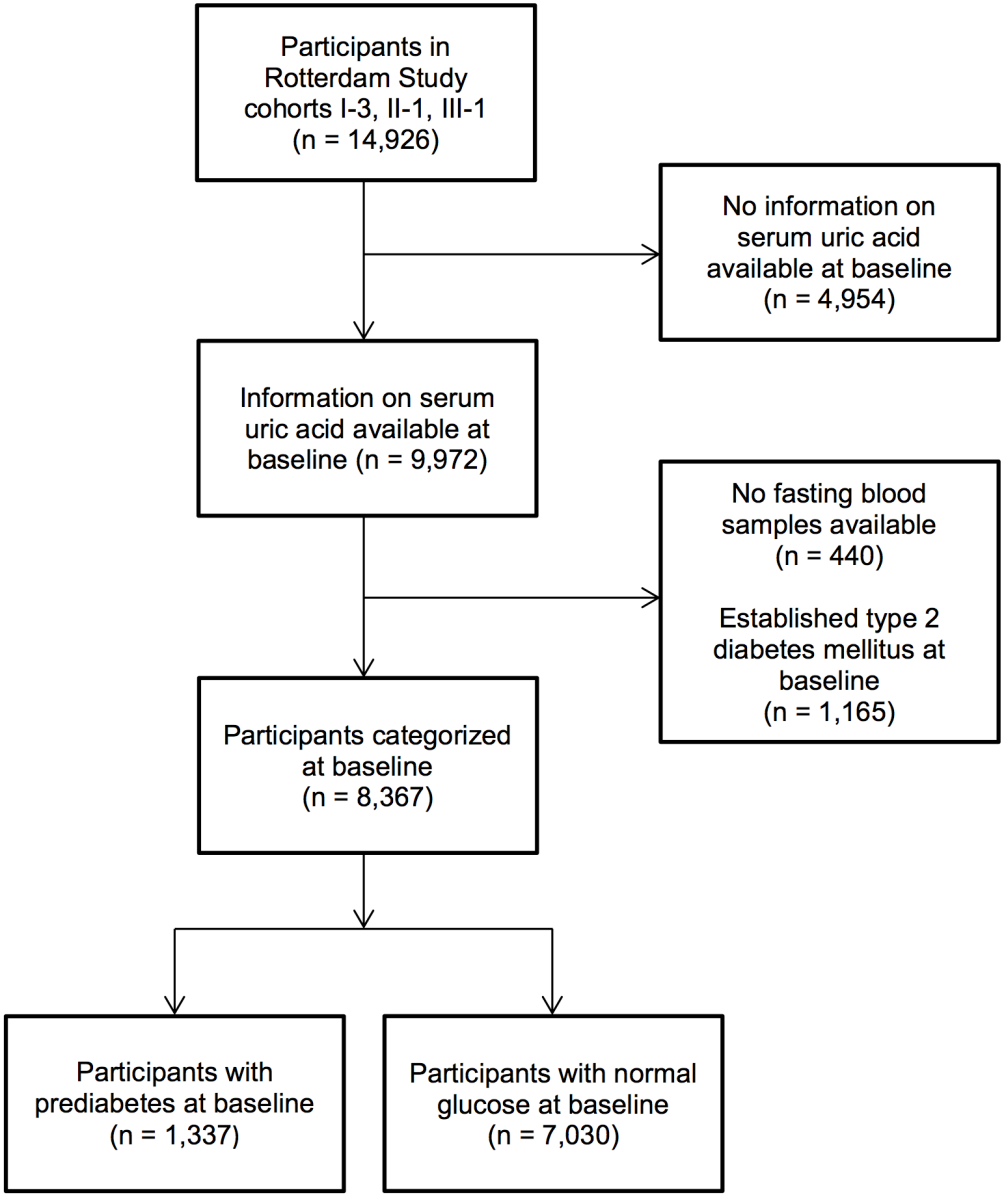
Selection of the study population.

The total study population eligible for analysis (n = 8,367) was divided into two mutually exclusive subgroups: a subgroup with normoglycaemia at baseline (n = 7,030) and a subgroup with prevalent prediabetes at baseline (n = 1,337). The selection procedure of our study population and the subgroups is outlined in Fig 1. Baseline characteristics of the study population are presented in Table 1.

**Table 1:** Baseline characteristics of the study population.

		Normoglycaemia at baseline n = 7,030	Missing data (%)	Prediabetes at baseline n = 1,337	Missing data (%)
Age (years)		64.2 (9.7)	0.0%	66.6 (9.4)	0.0%
Sex	Male (%) Female (%)	2,890 (41.1%) 4,140 (58.9%)	0.0%	665 (49.7%) 672 (50.3%)	0.0%
Body Mass Index		26.7 (3.9)	0.7%	28.5 (4.4)	0.6%
Serum Total Cholesterol (mmol/L)		5.8 (1.0)	0.0%	5.8 (1.0)	0.2%
Serum HDL (mmol/L)		1.4 (0.4)	0.6%	1.3 (0.4)	0.8%
Systolic Blood Pressure (mmHg)		137.2 (20.5)	0.5%	145.4 (20.8)	0.2%
Serum Insulin (pmol/L)^a^		66.0 (44.0)	0.2%	93.0 (67.0)	0.1%
Alcohol Consumption (g/day)^a^^b^		10.1 (12.6)	30.6%	13.7 (17.7)	35.2%
	Active (%) Former or Never (%)	6,008 (85.2%) 975 (14.1%)	0.7%	1,154 (86.3%) 176 (13.2%)	0.5%
Smoking	Active (%) Former or Never (%)	1,236 (17.6%) 5,794 (82.4%)	0.7%	237 (17.7%) 1,100 (82.3%)	0.4%
Hypertension^c^	Yes (%) No (%)	3,981 (56.6%) 3.049 (43.4%)	1.3%	1,000 (74.8%) 337 (25.2%)	0.7%
Use of diuretics	Yes (%) No (%)	581 (8.3%) 6,449 (91.7%)	2.9%	199 (14.9%) 1,138 (85.1%)	3.1%
Serum Glucose (mmol/L)^a^		5.3 (0.6)	0.0%	6.3 (0.4)	0.2%
Estimated Glomerular Filtration Rate (mL/min/1.73m^2^)		79.9 (15.7)	1.2%	77.4 (16.1)	0.5%
Metabolic Equivalents of Task (hours/week)^a^		71.6 (64.5)	11.6%	68.7 (64.0)	10.2%
Serum Uric Acid (mmol/L)		0.31 (0.07)	n/a	0.35 (0.08)	n/a

Variables are presented as mean (standard deviation) unless otherwise indicated.

^a^Variable is presented as median (interquartile range).

^b^Median alcohol consumption applies only to active drinkers.

^c^Hypertension is defined as having a systolic blood pressure > 140 mmHg, a diastolic blood pressure > 100 mmHg or receiving blood-pressure lowering medication with “hypertension” as an indication.

**Fig 2:**
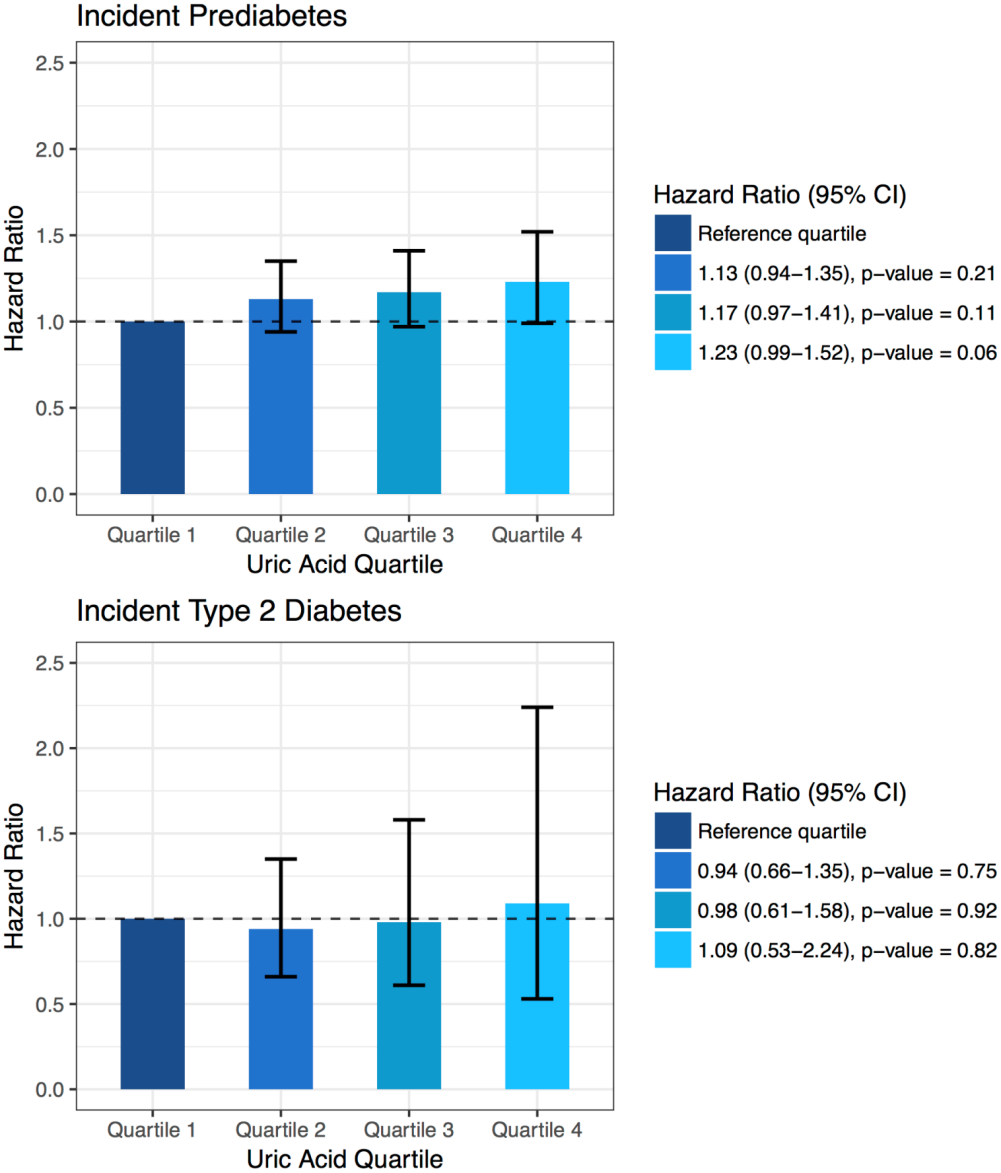
Quartile-specific hazard ratios for serum uric acid in association with incident prediabetes and incident type 2 diabetes mellitus.

Over a mean follow-up time of 7.5 years, 1,071 individuals with normoglycaemia at baseline developed prediabetes (incidence rate 20.2 per 1,000 person-years). In this analysis, the percentage of individuals who were lost to follow up was 0.6% (40 out of 7,030 individuals). The results of our analysis of the association between serum uric acid and incident prediabetes are presented in Table 2. We found a significant association between serum uric acid and incident prediabetes within individuals who were normoglycaemic at baseline in a model adjusted only for age, sex and cohort (HR 1.31 per SD increment, 95% confidence interval (CI) 1.23; 1.40). This association was attenuated but remained significant in the multivariable-adjusted model (HR 1.10, 95% CI 1.01; 1.18). Performing separate analyses for men and women, we found that the association between serum uric acid and incident prediabetes was present in both men (HR 1.28, 95% CI 1.16; 1.41) and women (HR 1.34, 95% CI 1.23; 1.45) in models adjusted for age and cohort (Table 3). After multivariable adjustment, serum uric acid was significantly associated with incident prediabetes among women (HR 1.13, 95% CI 1.02; 1.25) but not among men (HR 1.08, 95% CI 0.96; 1.21). Exclusion of individuals who use diuretics or individuals with hypertension did not materially change our findings (Table 4). The association failed to reach statistical significance upon exclusion of individuals with a BMI ≥ 25 (HR 1.14, 95% CI 0.98; 1.33). In the multivariable-adjusted model we also analysed serum uric acid in quartiles, providing quartile-specific HRs relative to the first quartile (Fig 2).

**Table 2:** The association between serum uric acid and incidence of prediabetes and type 2 diabetes mellitus.

	Incident prediabetes in normoglycaemic individuals	P-value	Incident type 2 DM in prediabetic individuals	P-value
Model 1^a^	1.31 (1.23; 1.40)	< 0.001	1.17 (1.06; 1.30)	0.002
Model 2^b^	1.30 (1.21; 1.40)	< 0.001	1.21 (1.08; 1.35)	0.001
Model 3^c^	1.10 (1.01; 1.18)	0.022	1.07 (0.94; 1.21)	0.330

Results are presented as Hazard Ratio (95% confidence interval) for a standard deviation increment in serum uric acid.

^a^Model 1: adjusted for age, sex and Rotterdam Study cohort.

^b^Model 2: model 1 + hypertension status, serum total cholesterol, eGFR, MET-hours per week, systolic blood pressure and use of diuretics.

^c^Model 3: model 2 + daily alcohol intake, serum HDL, smoking status, BMI, serum glucose and serum insulin.

**Table 3:** The association between serum uric acid and incidence of prediabetes and type 2 diabetes mellitus, stratified by gender.

	Model	Incident prediabetes in normoglycaemic individuals	P-value	Incident type 2 diabetes in prediabetic individuals	P-value
Men	1^a^	1.28 (1.16; 1.41)	< 0.001	1.19 (1.01; 1.40)	0.038
	2^b^	1.26 (1.13; 1.40)	< 0.001	1.30 (1.09; 1.56)	0.004
	3^c^	1.08 (0.96; 1.21)	0.216	1.23 (1.01; 1.48)	0.039
Women	1^a^	1.34 (1.23; 1.45)	< 0.001	1.18 (1.03; 1.35)	0.015
	2^b^	1.35 (1.23; 1.48)	< 0.001	1.19 (1.02; 1.38)	0.027
	3^c^	1.13 (1.02; 1.25)	0.024	1.00 (0.84; 1.19)	0.877

Results are presented as Hazard Ratio (95% confidence interval) for a standard deviation increment in serum uric acid.

^a^Model 1: adjusted for age, sex and Rotterdam Study cohort.

^b^Model 2: model 1 + hypertension status, serum total cholesterol, eGFR, MET-hours per week, systolic blood pressure and use of diuretics.

^c^Model 3: model 2 + daily alcohol intake, serum HDL, smoking status, BMI, serum glucose and serum insulin.

**Table 4:** Subgroup analyses for the association between serum uric acid and incident prediabetes and incident type 2 diabetes mellitus.

	Incident prediabetes in normoglycaemic individuals	P-value	Incident type 2 DM in prediabetic individuals	P-value
Exclusion of participants who use diuretics	1.11 (1.02; 1.21)	0.016	1.05 (0.92; 1.21)	0.497
Exclusion of participants with hypertension	1.16 (1.00; 1.34)	0.045	1.14 (0.84; 1.56)	0.412
Exclusion of participants with a BMI ≥ 25	1.14 (0.98; 1.33)	0.097	1.10 (0.75; 1.61)	0.647

Results are presented as multivariable-adjusted Hazard Ratios (95% confidence interval) for a standard deviation increment in serum uric acid, adjusted for age, sex, Rotterdam Study cohort, hypertension status, serum total cholesterol, eGFR, MET-hours per week, systolic blood pressure, use of diuretics, daily alcohol intake, serum HDL, smoking status, BMI, serum glucose and serum insulin.

A total of 407 individuals with prediabetes at baseline developed type 2 DM over a mean follow-up time of 7.2 years (incidence rate 42.4 per 1,000 person-years). In this analysis, the percentage of individuals who were lost to follow up was 0.4% (6 out of 1,337 individuals). Serum uric acid was significantly associated with incident type 2 DM in individuals with prediabetes in a model adjusting only for age, sex and cohort (HR 1.17, 95% CI 1.06; 1.30), but this association weakened and was not statistically significant in the multivariable-adjusted model (HR 1.07, 95% CI 0.94; 1.21) (Table 2). In sex-specific analyses, the association was significant among men (HR 1.19, 95% CI 1.01; 1.40), and women (HR 1.18, 95% CI 1.03; 1.35) in models adjusted for age and cohort (Table 3). After multivariable adjustment, serum uric acid was significantly associated with incident type 2 DM among men (HR 1.23, 95% CI 1.01; 1.48) but not among women (HR 1.00, 95% CI 0.84; 1.19). Exclusion of diuretic users, individuals with hypertension or individuals with a BMI ≥ 25 did not change our findings (Table 4). No significant difference was observed in any serum uric acid quartile compared to the first quartile (Fig 2).

## Discussion

We have found that higher serum uric acid levels are associated with an increased risk of incident prediabetes in individuals with normoglycaemia aged 45 years or over, independently of confounders. No significant association was observed between serum uric acid and incident type 2 DM in individuals with prediabetes after multivariable adjustment.

The result with relation to incident prediabetes is consistent with previous research on this subject using impaired fasting glucose as an endpoint.[14–17]. This could indicate that serum uric acid is more closely associated with early-phase rather than late-phase mechanisms that play a role in the development of type 2 DM. Typically, insulin resistance impairs pancreatic β-cell physiology and compensatory mechanisms, thereby inducing β-cell dysfunction as a consequence.[18] Insulin resistance could therefore be regarded as a reflection of early mechanisms that contribute to the development of type 2 DM, whereas β-cell dysfunction reflects the influence of late-stage mechanisms.[19] Currently, not much evidence is available concerning the relation between serum uric acid and pancreatic β-cell function. Tang and colleagues found an independent positive association between serum uric acid levels and residual pancreatic β-cell function.[20] In their cross-sectional analysis of 1,021 individuals with type 2 DM, they observed that patients with higher serum uric acid had greater insulin secretion ability in early disease stages, but their residual β-cell function decayed more quickly. The authors suggest that this increased insulin secretion might be a compensatory mechanism to overcome initial insulin resistance. In addition, Shimodaira and colleagues observed a significant negative association between serum uric acid and disposition index, a measure of pancreatic β-cell function, in a cross-sectional analysis among non-diabetic Japanese women after adjustment for age, BMI, systolic blood pressure, HbA1c, serum triglyceride level, serum HDL level and use of antihypertensive or antilipidemic drugs.[21] However, no definitive conclusions regarding the association between serum uric acid and pancreatic β-cell function can be drawn at this point. Further population-based, prospective studies investigating this association are warranted. Although the association between serum uric acid and incident prediabetes was not significant among individuals with BMI < 25, this finding is most likely due to a lack of statistical power, because individuals with a BMI ≥ 25 constitute over half of our sample size in this subgroup.

Serum uric acid has been investigated in relation to incident type 2 DM in individuals with impaired fasting glucose by Kramer and colleagues, who found a significant association (OR 1.75, 95% CI 1.1; 2.9) after adjusting for various confounders in study population with characteristics similar to ours.[22] We were not able to replicate this finding in our analysis, in which we had a considerably larger sample available and were able to adjust for a more comprehensive set of confounding variables. It is possible that residual confounding in the previous study could account for this difference, because Kramer and colleagues were unable to adjust for smoking status and serum HDL level. These covariates were particularly impactful in our multivariable-adjusted model. Excluding these covariates from the model yields an increase in the effect estimate (HR 1.13, 95% CI 1.00; 1.27) compared to the model which includes them (HR 1.07, 95% CI 0.94; 1.21). We also observe a steep decrease in the estimated hazard ratio for incident type 2 diabetes between model 2 and 3 in our analysis. The variable that is responsible for most of this decrease is serum HDL. It has been demonstrated that serum HDL is associated with plasma glucose levels and that it is strongly inversely associated with serum uric acid levels.[23, 24] Therefore, serum HDL can be regarded as a particularly strong confounder of this association.

There have been conflicting results reported in the literature about a possible sex-specific nature of the association between serum uric acid and impaired fasting glucose.[16, 17] In our study, we observe that serum uric acid is significantly associated with incident prediabetes among normoglycaemic women, but not among normoglycaemic men. Several studies report that the association between serum uric acid and glucose-related endpoints is especially pronounced among women.[15, 16, 25, 26] The difference between men and women with relation to incident prediabetes in our study can likely be attributed to residual confounding. We also have fewer events among men (n = 439) than among women (n = 632) in this analysis, which might lead to more imprecision in our estimated hazard ratio for men.

In contrast to this finding relating to incident prediabetes, serum uric acid was significantly associated with incident type 2 diabetes among men with prediabetes, but not among women with prediabetes in our study after multivariable adjustment. This observation was despite the fact that the number of events was higher among women (n = 222) than among men (n = 185) in this analysis. No other study has investigated the relation between serum uric acid and type 2 diabetes specifically among men with established glucose intolerance. Our result might suggest that serum uric acid affects women more strongly in the early stages of glucose intolerance development, whereas it affects men more strongly in more advanced stages. Potential biological mechanisms underlying this phenomenon have not yet been investigated in the literature, and further research is warranted.

Our findings build on the conclusion of a report by Kodama and colleagues, who performed a meta-analysis on the association between serum uric acid and incident type 2 diabetes in populations not stratified by glucose tolerance status (normoglycaemia or prediabetes) at baseline.[27] They conclude that serum uric acid is significantly associated with incident type 2 diabetes mellitus across 11 cohort studies, and that their result should encourage other studies to identify sub-populations for which the association might be especially important. We report that serum uric acid appears to be most strongly associated with the early stages of the development of type 2 diabetes. A similar meta-analysis by Jia and colleagues also found a positive association between serum uric acid and a combined endpoint of incident impaired fasting glucose and incident type 2 diabetes.[5] Our results further characterize the association between serum uric acid and glucose intolerance by treating incident prediabetes and incident type 2 diabetes as separate endpoints.

The strengths of our study include its prospective nature, which minimizes the chance of reverse causation, its long follow-up time and our ability to adjust for a large set of confounders. We provide a comprehensive overview of the relation between serum uric acid and different sub-stages on the spectrum between normoglycaemia and type 2 DM. However, our study population consisted of mainly elderly individuals and roughly 95% of our participants were of Caucasian ethnicity. Therefore, our results cannot be generalized to populations with a different composition without further consideration. Finally, we cannot exclude the possibility of residual confounding, although the fact that we adjusted for many covariates should minimize the chance of this type of bias.

In conclusion, serum uric acid was independently and positively associated with incident prediabetes in individuals with normoglycaemia but not with incident type 2 DM in individuals with prediabetes in a large population-based cohort of individuals aged 45 years and over. Our results indicate that serum uric acid might be more closely associated with early-phase pathogenic mechanisms that contribute to the development of type 2 DM rather than late-phase mechanisms.
